# One-year health status outcomes of unstable angina versus myocardial infarction: a prospective, observational cohort study of ACS survivors

**DOI:** 10.1186/1471-2261-7-28

**Published:** 2007-09-12

**Authors:** Thomas M Maddox, Kimberly J Reid, John S Rumsfeld, John A Spertus

**Affiliations:** 1 Denver VAMC/University of Colorado Health Science Center, Denver, CO, USA; 2 Mid America Heart Institute of Saint Luke's Hospital, Kansas City, MO, USA; 3 University of Missouri – Kansas City, Kansas City, MO, USA

## Abstract

**Background:**

Unstable angina (UA) patients have lower mortality and reinfarction risks than ST-elevation (STEMI) or non-ST elevation myocardial infarction (NSTEMI) patients and, accordingly, receive less aggressive treatment. Little is known, however, about the health status outcomes (angina, physical function, and quality of life) of UA versus MI patients among survivors of an ACS hospitalization.

**Methods:**

In a cohort of 1,192 consecutively enrolled ACS survivors from two Kansas City hospitals, we evaluated the associations between ACS presentation (UA, NSTEMI, and STEMI) and one-year health status (angina, physical functioning and quality of life), one-year cardiac rehospitalization rates, and two-year mortality outcomes, using multivariable regression modeling.

**Results:**

After multivariable adjustment for demographic, hospital, co-morbidity, baseline health status, and treatment characteristics, UA patients had a greater prevalence of angina at 1 year than STEMI patients (adjusted relative risk [RR] = 1.42; 95% CI [1.06, 1.90]) and similar rates as NSTEMI patients (adjusted RR = 1.1; 95% CI [0.85, 1.42]). In addition, UA patients fared no better than MI patients in Short Form-12 physical component scores (UA vs. STEMI score difference -0.05 points; 95% CI [-2.41, 2.3]; UA vs. NSTEMI score difference -1.91 points; 95% CI [-4.01, 0.18]) or Seattle Angina Questionnaire quality of life scores (UA vs. STEMI score difference -1.39 points; 95% CI [-5.63, 2.85]; UA vs. NSTEMI score difference -0.24 points 95% CI [-4.01, 3.54]). Finally, UA patients had similar rehospitalization rates as MI patients (UA vs. STEMI adjusted hazard ratio [HR] = 1.31; 95% CI [0.86, 1.99]; UA vs. NSTEMI adjusted HR = 1.03; 95% CI [0.73, 1.47]), despite better 2-year survival (UA vs. STEMI adjusted HR = 0.51; 95% confidence interval (CI) [0.28, 0.95]; UA vs. NSTEMI adjusted HR = 0.40; 95% CI [0.24, 0.65]).

**Conclusion:**

Although UA patients have better survival rates, they have similar or worse one-year health status outcomes and cardiac rehospitalization rates as compared with MI patients. Clinicians should be aware of the adverse health status outcome risks for UA patients and consider close monitoring for the opportunity to improve their health status and minimize the need for subsequent rehospitalization.

## Background

Acute coronary syndrome (ACS) clinical presentations, including ST-elevation myocardial infarction [STEMI], non-ST-elevation myocardial infarction [NSTEMI], and unstable angina [UA], are associated with different mortality and recurrent myocardial infarction (MI) rates [1-4]. Accordingly, optimal ACS management, as outlined in the American College of Cardiology/American Heart Association guidelines, stratifies and treats patients differently according to their presentation so that those with the greatest mortality risk receive the most aggressive therapy [1,5-8]. However, mortality and rehospitalization are not the only clinically important outcomes. Patient health status outcomes, including their symptom burden, functional status, and health-related quality of life, are critical outcomes from the patient's perspective [9-12]. Accordingly, patient health status has recently been advocated as a marker of healthcare quality [13-15].

Despite its clinical importance, little is known about the association between ACS presentation and health status outcomes, especially among UA patients. Given that many cardiac patients are initially identified during an ACS presentation, characterizing this association from the perspective of this presentation is essential to better prognosticate and treat patients with symptomatic coronary disease. Accordingly, we evaluated one-year health status outcomes in a consecutive cohort of ACS patients as a function of their clinical presentation. Identifying patients at risk for poorer health status could identify the need for improved methods of risk stratification so as to improve care and outcomes in ACS patients.

## Methods

### Study population and design

**Figure 1:**
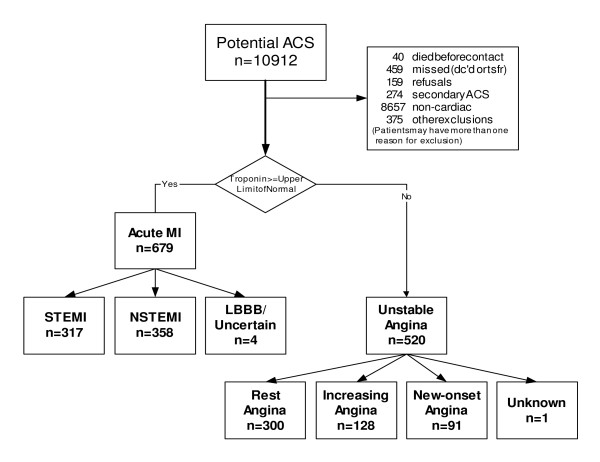
Flowchart of screened and enrolled patients in the INFORM registry.

The Investigation oF Outcomes from acute coRonary syndroMes (INFORM) registry is a prospective, observational cohort study of consecutively hospitalized ACS patients at two Kansas City hospitals, the Mid America Heart Institute and Truman Medical Center, to identify determinants of health status outcomes among ACS survivors. It was powered for minimal detectable differences of >5 points in SAQ quality of life (disease perception) and angina frequency scores. In total, 10,911 consecutive hospitalized patients who had a troponin blood test performed at either hospital between March 2001 and October 2002 were prospectively screened for a possible ACS. 1199 patients with confirmed ACS (see definitions below) were enrolled in the registry (Figure 1) and underwent detailed interviews and chart abstractions to obtain their socio-demographic, health status, clinical, and treatment characteristics. All data elements conformed to the standards established in the American College of Cardiology Task Force on Clinical Data Standards[16]. Since our investigation focused on those who survived to hospital discharge, those patients who died during hospitalization (n = 7; 3 STEMI, 3 NSTEMI, and 1 UA) were excluded from the analyses. One year after their index hospitalization, follow-up phone interviews were conducted to collect health status and rehospitalization information. Each participant signed an informed consent to participate in this study and Institutional Review Board approval from both institutions (Saint Luke's Health System Institutional Review Board and the University of Missouri-Kansas City Adult Health Sciences Institutional Review Board) was obtained prior to the conduct of the study.

### Acute coronary syndrome classification

Standard definitions were used to confirm patients' ACS diagnosis [17]. STEMI patients presented with suggestive cardiac symptoms, diagnostic electrocardiogram (EKG) changes (ST segment elevation or new-onset left bundle branch block [LBBB]), and a positive troponin blood test (during the course of this investigation, the following assays and thresholds were used by the enrolling centers: cTroponin-I assay >0.15 ng/mL, Advia-Centaur, Bayer Diagnostics, Tarrytown, NY; cTroponin-I assay >0.05 ng/mL, Dade Dimension RXL, Dade Behring Diagnostics, Deerfield, IL; cTroponin-I assay >1.9 ng/mL, Abbott Labs, Abbott Park, IL). NSTEMI patients presented with suggestive cardiac symptoms and/or EKG changes (e.g. ST segment depressions and/or T wave changes), and a positive troponin blood test. UA patients presented with suggestive cardiac symptoms, as defined by at least one of the following: new onset angina (<2 months) of at least Canadian Cardiovascular Society Classification (CCSC) class III, prolonged (>20 minutes) rest angina, recent (<2 months) worsening of angina, or angina that occurred within 2 weeks of a previous MI [18]. Although EKG changes were not a requirement for diagnosis, nearly one-half of UA patients had ischemic EKG changes on admission (LBBB 4%, ST-elevations 9%, ST-depressions 12%, T-wave inversions 22%). By definition, all UA patients had a negative troponin blood test. To further increase the specificity of the unstable angina diagnosis, those patients with a diagnostic study that excluded obstructive coronary disease, cardiac perfusion defects, or segmental wall motion abnormalities (e.g. coronary angiography, nuclear or echocardiographic stress testing) (n = 125) or confirmed an alternative explanation for their presentation (e.g. esophagogastroduodenoscopy) were excluded. Three physicians reviewed the charts of all patients for whom diagnostic uncertainty (n = 45) remained and attained consensus on the final diagnosis.

### Health status, rehospitalization, and mortality assessment

After excluding expired patients by querying the Social Security Death Master File, surviving patients underwent follow-up telephone interviews to obtain one-year rehospitalization rates and health status outcomes. Health status outcomes were measured using the Seattle Angina Questionnaire (SAQ) and the Short Form-12 Version 2 (SF-12). The SAQ is a 19-item disease-specific health status measure for patients with coronary artery disease (CAD) that has well-established validity, reliability, sensitivity to clinical change, and prognostic value [13,19-22]. Five domains are assessed: anginal frequency, anginal stability, physical limitation, treatment satisfaction, and disease perception. The scales used in these analyses range from 0–100, where higher scores indicate fewer symptoms and higher quality of life. The SF-12 is a generic health status measure that is converted into physical and mental component scores [23]. A score of 50 reflects the United States' population mean, and a deviation of 10 points reflects 1 standard deviation from that mean. Higher scores indicate better physical and mental functioning.

We measured the health status outcomes of symptoms, physical functioning, and health-related quality of life with the SAQ angina frequency scale, the SF-12 physical component scale, and the SAQ disease perception scale, respectively. The SAQ scales of treatment satisfaction and angina stability were less informative to our research question, and thus not analyzed. In addition, the SF-12 physical functioning scale was used instead of the SAQ physical limitation scale because the SF-12 scale was more representative of activities that registry patients performed and the disease-specific SAQ created more missing data for physical function than did the SF-12. One-year cardiac rehospitalization rates were collected by patient self-report at the follow-up interview. Hospitalizations for chest pain, heart failure, myocardial infarction (MI), cardiac revascularization (percutaneous coronary intervention [PCI], or coronary artery bypass graft [CABG]) were defined as cardiac. Hospitalizations for other reasons were coded as non-cardiac. Two-year mortality data was determined by querying the Social Security Administration Death Master File. A final query of the Social Security Death Master File, hospital and outpatient records for vital status and hospitalization data was performed in October 2004.

### Statistical analysis

After categorizing patients by their ACS presentation (STEMI, NSTEMI and UA), baseline clinical characteristics were compared. Categorical data were reported as frequencies, and differences were assessed using chi-square tests. Continuous data were reported as means ± standard deviations and differences were assessed using analysis of variance.

To evaluate the independent association between ACS presentation and outcomes, multivariable models were created to adjust for all other differences in socio-demographic (age, race, sex, and insurance status), hospital (Mid-America Heart Institute or Truman Medical Center), clinical (prior MI, prior PCI, prior CABG, congestive heart failure, hypertension, diabetes, hyperlipidemia, cerebrovascular accident/transient ischemic attack, renal failure, anemia, and tobacco use), baseline physical function or quality of life (SF-12 physical functioning scores or SAQ quality of life scores) and treatment (both revascularization (PCI, CABG, or thrombolysis) and discharge medications (angiotensin converting enzyme [ACE] inhibitors, lipid lowering agents, beta-blockers, calcium channel blockers, nitrates, and aspirin)) characteristics. Anemia was defined as hemoglobin values less than the fifth percentile of the sex, race, and age-matched population [24]. Categories of variables (socio-demographic, site, clinical, baseline health status, and treatment) were entered sequentially into the model and presented accordingly, in order to illustrate the additive effects of each variable group on the various outcomes.

SAQ angina frequency scores were modeled as a dichotomous outcome of any angina vs. no angina at one year after index ACS hospitalization because of the skewed nature of the measure and our clinical goal of seeking to completely eliminate patients' angina [25]. Since one-year angina in our study was a relatively frequent event, we estimated adjusted relative risks directly using a modified Poisson regression model, rather than using logistic regression which estimates adjusted odds ratios [26]. SF-12 physical component and SAQ quality of life scores were modeled as continuous variables using multivariable linear regression. Model diagnostics were conducted using residual and normal probability plots.

Unadjusted differences in mortality and rehospitalization rates between ACS classes were described graphically with Kaplan-Meier survival plots and compared using the log-rank test. Adjusted differences in mortality and rehospitalization rates between ACS classes, using the same covariates as the health status models, were compared using multivariable Cox proportional hazards regression models. Cox proportional hazards assumptions were tested using Schoenfeld residuals.

All analyses were performed with SAS version 9.3.1 (SAS Institute, Inc., Cary, NC) and R version 2.1.1 (R Development Core Team. R: A Language and Environment for Statistical Computing. Vienna, Austria). Statistical significance for all analyses was assumed when the p value was less than 0.05. Scheffe correction techniques for multiple comparisons were used for pair-wise comparisons.

### Missing data

Among the 1192 patients who survived until hospital discharge, 77 (6.5%) died within 12 months of follow-up. Health status outcomes were not imputed for those who died. Thus, our health status results characterize those who survived for at least one year after discharge. Among survivors, 210 patients (17.6%) were not interviewed at one year, because they could not be contacted (73% of non-respondents), or refused to complete the interview (27% of non-respondents). Among those lost-to-follow-up patients, there were no significant differences by ACS presentation.

We explored potential selection biases in the rehospitalization and health status outcomes through the use of sensitivity and propensity model analyses. Sensitivity analysis was performed by imputing poor health status scores to those patients (n = 22) who were too ill to participate in the follow-up interview. Specifically, patients were assigned a SAQ Angina Frequency score indicating presence of angina, an SF-12 physical functioning score of 25, and a SAQ quality of life score of 50; the lowest deciles for each outcome. Regression analyses were repeated using these imputed values and no statistically or clinically significant differences in results were noted (results not shown).

As an alternative approach for handling missing health status data from patients who refused 1-year interviews or could not be contacted, propensity scores were computed using non-parsimonious multivariable logistic regression models to predict the likelihood of unsuccessful follow-up [27]. Predictor variables included all available demographic, socio-economic and lifestyle factors, clinical characteristics, vital signs and laboratory studies, disease severity, baseline health status, medication, acute, and non-acute treatments received during patients' initial ACS hospitalization. From these models, a probability of success for completing an interview was calculated. The reciprocal of this probability was then assigned to those patients' scores in the multivariable regression analyses in order to assess for potential observable bias from those lost to follow-up by weighting patients that are similar to those with missing data more heavily [27]. These analyses also demonstrated that the missing patients did not impact our primary findings. In light of the consistency of our findings with both imputation and propensity approaches for handling missing data, we have presented only the primary results.

## Results

### Patient characteristics by ACS presentation

Among the 1192 ACS survivors, 318 (27%) presented with STEMI, 355 (30%) with NSTEMI, and 519 (44%) with UA. Table 1 illustrates the socio-demographic and clinical characteristics, baseline health status scores, revascularization therapies and discharge medications for each ACS class. Demographically, NSTEMI patients were more likely to be older than either STEMI or UA patients, STEMI patients were more likely to be male than either NSTEMI or UA patients, and STEMI patients were more likely to be white compared to UA patients. Clinically, UA patients had significantly higher rates of prior cardiac disease, as indicated by prior myocardial infarction and revascularization, as compared with both STEMI and NSTEMI patients. In addition, UA patients had significantly worse physical functioning and poorer quality of life on admission than either STEMI or NSTEMI patients.

**Table 1:** Demographic and clinical characteristics by ACS presentation

**Variables**	**STEMI**	**NSTEMI**	**UA**	**ANOVA**
	**n = 321**	**n = 358**	**n = 520**	**p-value**
**Demographics**				
Age	60.8 +/- 12.5	63.2 +/- 13.0 *	61.0 +/- 13.0 ‡	p = 0.023
Male	70.7%	56.7% *	59.6% †	p < 0.001
Caucasian	85.3%	81.6%	76.5% †	p = 0.08
Insured	84.4%	89.4%	85.6%	p = 0.132
Employed (full or part-time)	46.3%	36.9% *	34.6% †	p = 0.02
				
**Past medical history**				
History of myocardial infarction	22.1%	24.3%	44.4% †‡	p < 0.001
History of percutaneous coronary intervention	21.8%	27.7%	46.2% †‡	p < 0.001
History of coronary artery bypass graft	10.0%	18.4% *	25% †‡	p < 0.001
Congestive heart failure	3.7%	7.8% *	8.5% †	p = 0.027
Hypertension	56.4%	63.4%	72.7% †‡	p < 0.001
Diabetes mellitus	19.6%	24.6%	32.7% †‡	p < 0.001
Hyperlipidemia	51.1%	58.1%	68.3% †‡	p < 0.001
Transient ischemic attack	1.2%	1.7%	2.1%	p = 0.65
Cerebrovascular accident	0.9%	2.5%	2.7%	p = 0.208
Chronic obstructive pulmonary disease/asthma	5.6%	12.8% §	12.9% †	p = 0.001
Renal failure	0.9%	2.0%	2.7%	p = 0.214
Anemia	62.2%	61.7%	50.2%	p < 0.001
Tobacco use	68.2%	69.9%	73.2% †‡	p < 0.001
				
**Baseline health status**				
SAQ angina rates	95.0%	87.4% *	93.1% ‡	p < 0.001
SF-12 physical component scores	42.7 +/- 11.4	39.1 +/- 12.3 *	35.3 +/- 11.9 †‡	p < 0.001
SAQ quality of life scores	50.4 +/- 17.3	53.4 +/- 20.2 *	47.0 +/- 18.8 †‡	p < 0.001
				
**ACS therapies**				
Primary reperfusion	86.3%	65.1% *	42% †‡	p < 0.001
Percutaneous coronary intervention	63.9%	62% §	40.8% †‡	p < 0.001
Lysis	22.4%	3.1% §	1.2% †‡	p < 0.001
Coronary artery bypass graft	4.4%	3.9%	2.9%	p < 0.001
				
**Discharge meds**				
Angiotensin converting enzyme inhibitors	18.1%	30.2% *	36.5% †‡	p < 0.001
Lipid lowering	79.8%	77.7%	75.2%	p = 0.191
Beta-blockers	87.5%	84.4%	72.5% †‡	p < 0.001
Calcium channel blockers	10.0%	14.2%	23.7% †‡	p < 0.001
Nitrates	9.3%	12.0%	18.1% †‡	p < 0.001
Acetylsalicylic acid (aspirin)	95.9%	93.9%	92.7%	p = 0.379

*STEMI v NSTEMI p-value < 0.05; †STEMI v UA p-value < 0.05; ‡NSTEMI v UA p-value < 0.05; ANOVA, analysis of variance; NSTEMI, non-ST-elevation myocardial infarction; STEMI, ST-elevation myocardial infarction; UA, unstable angina; SAQ, Seattle Angina Questionnaire; SF-12, Short Form 12

Treatments also differed by ACS classification. STEMI patients had the highest revascularization rates and UA patients the lowest (86.3% and 42.0%, respectively). Significant differences in discharge medication prescriptions were also observed. STEMI and NSTEMI patients were more likely to be treated with beta-blockers at discharge, while UA patients received higher rates of ACE-inhibitors, calcium-channel blockers, and nitrates (Table 1).

### Mortality and rehospitalization outcomes

**Figure 2:**
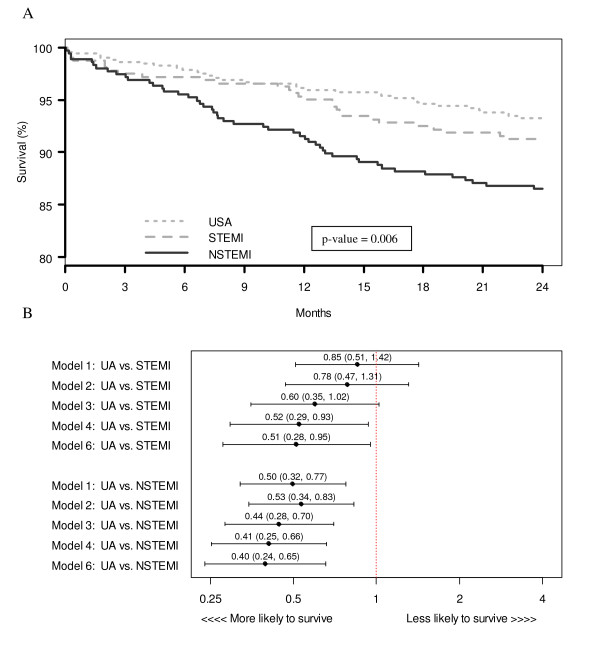
(a) Unadjusted Kaplan-Meier survival curves of two-year mortality by ACS presentation (b) Unadjusted and sequential adjustment of two-year mortality by ACS presentation (Model 1 = unadjusted comparison; Model 2 = adjustment for demographic variables (age, race, sex, insurance status); Model 3 = adjustment for demographic and hospital site variables (Mid-America Heart Institute or Truman Medical Center); Model 4 = adjustment for demographic, site, and clinical variables (prior angina, prior myocardial infarction, prior percutaneous coronary intervention, prior coronary artery bypass graft, congestive heart failure, hypertension, diabetes, hyperlipidemia, cerebrovascular accident/transient ischemic attack, renal failure, anemia and tobacco use); Model 6 = adjustment for demographic, site, clinical, and treatment (revascularization [percutaneous coronary intervention, coronary artery bypass graft, thrombolysis] and discharge medications [angiotensin converting enzyme inhibitors, lipid lowering agents, beta-blockers, calcium channel blockers, nitrates, and aspirin]) variables)

**Figure 3:**
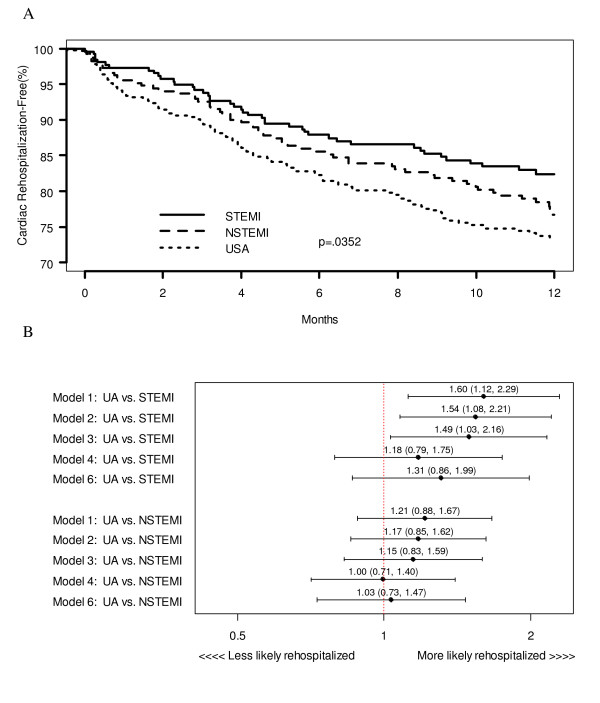
(a) Unadjusted Kaplan-Meier survival curves of one-year cardiac rehospitalization by ACS presentation (b) Unadjusted and sequential adjustment of one-year cardiac rehospitalization by ACS presentation (Model 1 = unadjusted comparison; Model 2 = adjustment for demographic variables (age, race, sex, insurance status); Model 3 = adjustment for demographic and hospital site variables (Mid-America Heart Institute or Truman Medical Center); Model 4 = adjustment for demographic, site, and clinical variables (prior angina, prior myocardial infarction, prior percutaneous coronary intervention, prior coronary artery bypass graft, congestive heart failure, hypertension, diabetes, hyperlipidemia, cerebrovascular accident/transient ischemic attack, renal failure, anemia and tobacco use); Model 6 = adjustment for demographic, site, clinical, and treatment (revascularization [percutaneous coronary intervention, coronary artery bypass graft, thrombolysis] and discharge medications [angiotensin converting enzyme inhibitors, lipid lowering agents, beta-blockers, calcium channel blockers, nitrates, and aspirin]) variables)

UA patients had the lowest two-year mortality rates, but similar one-year rehospitalization rates. At two years, 25 (7.9%) STEMI patients and 45 (12.8%) NSTEMI patients had died as compared with only 35 (6.7%) UA patients (log-rank p-value 0.006; Figure 2a). After multivariable analysis controlling for socio-demographic, site, clinical, and treatment characteristics, UA patients had significantly lower mortality rates compared to STEMI and NSTEMI patients (UA vs. STEMI hazard ratio (HR) = 0.51; 95% confidence interval (CI) [0.28, 0.95]; UA vs. NSTEMI HR = 0.40; 95% CI [0.24, 0.65]) (Figure 2b). At one year, 102 (26.6%) UA patients were rehospitalized for cardiac causes, as compared to 43 (17.6%) STEMI patients and 60 (23.3%) NSTEMI patients (log-rank p-value 0.035: Figure 3a). After multivariable adjustment, UA patients had similar one-year cardiac rehospitalization rates to STEMI and NSTEMI patients (UA vs. STEMI HR = 1.31; 95% CI [0.86, 1.99]; UA vs. NSTEMI HR = 1.03; 95% CI [0.73, 1.47]; Figure 3b).

### Health status outcomes

**Figure 4:**
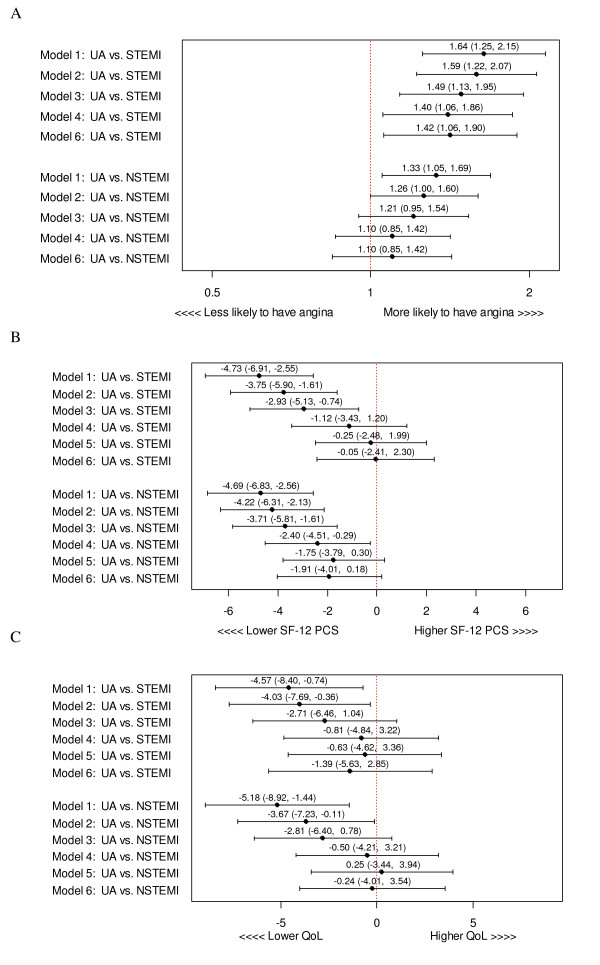
(a) Unadjusted and sequential adjustment of one-year angina by ACS presentation (b) Unadjusted and sequential adjustment of one-year physical functioning by ACS presentation (c) Unadjusted and sequential adjustment of one-year quality of life by ACS presentation (Model 1 = unadjusted comparison; Model 2 = adjustment for demographic variables (age, race, sex, insurance status); Model 3 = adjustment for demographic and hospital site variables (Mid-America Heart Institute or Truman Medical Center); Model 4 = adjustment for demographic, site, and clinical variables (prior angina, prior myocardial infarction, prior percutaneous coronary intervention, prior coronary artery bypass graft, congestive heart failure, hypertension, diabetes, hyperlipidemia, cerebrovascular accident/transient ischemic attack, renal failure, anemia and tobacco use); Model 5 = adjustment for demographic, site, clinical, and baseline health status variables (SF-12 physical component score and SAQ quality of life score models only); Model 6 = adjustment for demographic, site, clinical, baseline health status (SF-12 physical component score and SAQ quality of life score models only), and treatment (revascularization [percutaneous coronary intervention, coronary artery bypass graft, thrombolysis] and discharge medications [angiotensin converting enzyme inhibitors, lipid lowering agents, beta-blockers, calcium channel blockers, nitrates, and aspirin]) variables)

One year after discharge, UA patients had worse unadjusted angina rates, physical component scores, and quality of life scores than either STEMI or NSTEMI patients (Figure 4a–c). After sequential multivariable adjustment for all covariates, UA patients were more likely to experience angina than STEMI patients (UA vs. STEMI angina relative risk (RR) = 1.42; 95% CI [1.06, 1.90]). Adjusted angina rates between UA and NSTEMI were similar (UA vs. NSTEMI angina RR = 1.10; 95% CI [0.85, 1.42]) (Figure 4a), as were the remainder of health status scores between all three ACS classes (UA vs. STEMI adjusted mean physical component score difference = -0.05 points, 95% CI [-2.41, 2.30]; UA vs. NSTEMI adjusted mean physical component score difference = -1.91 points, 95% CI [-4.01, 0.18]; UA vs. STEMI adjusted mean quality of life score difference = -1.39 points, 95% CI [-5.63, 2.85]; UA vs. NSTEMI adjusted mean quality of life score difference = -0.24 points, 95% CI [-4.01, 3.54]) (Figure 4b,c).

## Discussion

Although previous studies have documented higher mortality among MI patients as compared to those with UA [1-3], none have systematically evaluated the association of ACS presentation with health status outcomes. Our study is the first to illustrate that UA patients have a higher prevalence of angina at 1 year than STEMI patients, even after adjustment for numerous differences in patient characteristics and treatment. Furthermore, we found that, in contrast to their better prognosis in terms of survival, UA patients had a similar prevalence of angina as compared with NSTEMI patients and similar one-year physical functioning scores, quality of life scores, and cardiac rehospitalization rates to both STEMI and NSTEMI patients. Our study suggests that since UA patients' favorable prognosis with respect to mortality does not translate to health status outcomes, close follow-up and monitoring of UA patients is needed.

With the recognition of MI patients' elevated mortality risk, the medical community substantially reorganized itself, resulting in community interventions to accelerate the recognition and treatment of potential MIs, emergency department chest pain centers for rapid patient triage, increased access to primary PCI, early institution of anti-platelet treatments and invasive risk stratification, resulting in impressive mortality reductions over the past decades [4,7,28-33]. However, the results of this study highlight the adverse health status outcome risks present in the UA population, as compared with MI patients. Given that these are critical outcomes for patients and providers alike, clinicians should be aware of these risks and should develop interventions to improve the health status outcomes of UA patients.

One previous report has also noted poorer health status outcomes among UA patients. Rumsfeld and colleagues evaluated 2733 ACS patients, using a general health status instrument, the Short Form-36 (SF-36), and followed patients for 7 months after their index hospitalization. They found that a discharge diagnosis of UA, as compared with MI, was associated with a worse SF-36 physical component score [34]. Our study strengthens and extends these initial findings by using a cardiac disease-specific instrument, assessing a broader range of health status outcomes (including angina and health-related quality of life in addition to physical functioning), adjusting for a greater number of potential confounders, and conducting observations over a longer follow-up period.

Several characteristics of the UA population may partially explain their worse angina outcomes. The high prevalence of pre-existing CAD and low revascularization rates among UA patients may contribute to their higher angina rates and lower health status scores, though adjustment for these factors did not eradicate the higher angina rates of UA patients compared to STEMI patients [35,36]. Other unmeasured characteristics of their care, such as less intensive outpatient medical and/or revascularization therapies, decreased medical follow-up, and decreased medication adherence among UA patients, may affect patients' long-term health status and represent important opportunities to improve their outcomes. Further research is needed to evaluate the outpatient care of ACS patients so that greater insights into the potential opportunities to improve the angina rates among UA patients may be uncovered.

Multiple studies have demonstrated improvements in anginal symptoms and subsequent health status among CAD patients with both medical therapy and revascularization, though most studies document greater health status improvements with revascularization strategies as compared to standard medical therapy [37-42]. Mortensen and colleagues noted improved three-year angina rates and SF-36 physical functioning scores among patients with inducible post-infarction ischemia who underwent invasive revascularization [37]. Similarly, Kim and colleagues demonstrated improved one-year angina rates and higher one-year quality of life scores among NSTEMI/UA patients randomized to an invasive revascularization treatment strategy [42]. Given the success of these strategies in improving health status outcomes among MI and stable CAD patients, randomized studies of these and other strategies on health status outcomes among UA patients should be considered. Concurrently, integration of objective health status assessment into the care of ACS patients could offer a tool to identify patients with residual symptoms and to potentially indicate a need for additional therapy to improve UA patients' rehospitalization rates and quality of life.

Our study has several potential limitations. First, it was conducted at only 2 Midwestern centers, which may limit the generalizability of our findings to other centers. Second, despite follow-up rates greater than 80%, missing data can potentially introduce bias. Although our sensitivity and propensity analyses suggested no important differences, we cannot rule out the possibility of unmeasured confounding from those patients without follow-up. Third, cardiac rehospitalization data was assessed by patient self-report, and thus subject to potential misclassification and recall bias. However, trained data collectors conducted phone interviews, thus minimizing this risk. Finally, we did not have detailed data about the outpatient treatment regimens of our patients, including the frequency of follow-up medical visits or their medication use over the year after discharge. Further research will need to define whether the intensity or quality of post-discharge care differs across the spectrum of ACS patients.

## Conclusion

In conclusion, we found that UA patients had a higher prevalence of angina than STEMI patients and similar physical functioning, quality of life, and cardiac rehospitalization outcomes as compared with both STEMI and NSTEMI patients one year after ACS presentation. Since multiple treatments, including medications and revascularization, are available to improve patients' angina, these findings identify an important potential opportunity for improving the quality of care for UA patients. Future studies to evaluate the impact of these interventions specifically in UA patients are needed. Until then, clinicians should remain as vigilant for persistent angina, functional limitations and poor quality of life among UA patients as they are among MI patients.

## Abbreviations

ACS, acute coronary syndrome; STEMI, ST-segment elevation myocardial infarction; NSTEMI, non-ST-segment elevation myocardial infarction; UA, unstable angina

## Competing interests

Dr. Spertus discloses he has leadership responsibilities for CV Outcomes, Inc., Health Outcomes Sciences and Outcomes Instruments; is a consultant for Amgen, United Healthcare, and Otsuka; receives research grant support from the National Institutes of Health, Amgen, Lilly, Roche Diagnostics, and the American College of Cardiology-National Cardiovascular Data Registry (ACC-NCDR); and owns the copyrights for the Seattle Angina Questionnaire, the Kansas City Cardiomyopathy Questionnaire, and the Peripheral Artery Questionnaire. The other authors declare that they have no competing interests.

## Authors' contributions

TMM and JAS conceived of the study, participated in its design and coordination, and drafted the manuscript. KJR performed the statistical analysis and assisted with manuscript drafts. JSR assisted with manuscript drafts and provided critical input. All authors read and approved the final manuscript.

## Pre-publication history

The pre-publication history for this paper can be accessed here:


